# RPD3 histone deacetylase and nutrition have distinct but interacting effects on *Drosophila* longevity

**DOI:** 10.18632/aging.100856

**Published:** 2015-12-08

**Authors:** Stewart Frankel, Jared Woods, Tahereh Ziafazeli, Blanka Rogina

**Affiliations:** ^1^ Department of Biology, University of Hartford, West Hartford, CT 06117, USA; ^2^ Department of Genetics & Genome Sciences, School of Medicine, University of Connecticut Health, Farmington, CT 06030, USA; ^3^ Institute for Systems Genomics, School of Medicine, University of Connecticut Health, Farmington, CT 06030, USA; ^4^ Current address: Division of Pediatric Endocrinology, Department of Pediatrics, Faculty of Health Sciences, McMaster University, Ontario, Canada

**Keywords:** Rpd3, dietary restriction, longevity, Drosophila, aging

## Abstract

Single-gene mutations that extend longevity have revealed regulatory pathways related to aging and longevity. RPD3 is a conserved histone deacetylase (Class I HDAC). Previously we showed that *Drosophila rpd3* mutations increase longevity. Here we tested the longevity effects of RPD3 on multiple nutrient levels. Dietary restriction (DR) has additive effects on RPD3-mediated longevity extension, but the effect may be modestly attenuated relative to controls. RPD3 and DR therefore appear to operate by distinct but interacting mechanisms. Since RPD3 regulates transcription, the mRNA levels for two proteins involved in nutrient signaling, 4E-BP and Tor, were examined in *rpd3* mutant flies. *4E-BP* mRNA was reduced under longevity-increasing conditions. Epistasis between RPD3 and 4E-BP with regard to longevity was then tested. Flies only heterozygous for a mutation in *Thor*, the *4E-BP* gene, have modestly decreased life spans. Flies mutant for both *rpd3* and *Thor* show a superposition of a large RPD3-mediated increase and a small *Thor*-mediated decrease in longevity at all food levels, consistent with each gene product having distinct effects on life span. However, DR-mediated extension was absent in males carrying both mutations and lessened in females. Our results support the view that multiple discrete but interacting mechanisms regulate longevity.

## INTRODUCTION

Single gene mutations that extend life span have provided important information about pathways that regulate aging and longevity [1]. A significant number of these mutations are in genes and pathways that are conserved between invertebrates and vertebrates, with the caveat that the pathways are often more complex in vertebrates. Many pathways implicated in longevity extension are related in some way to nutrient sensing or nutrient utilization, such as insulin signaling, energy production, and protein biosynthesis [1]. For example, the Tor pathway integrates signals from nutrient uptake, stress, growth factors, and cellular energy status [2]. Genetic manipulation of the Tor gene has been shown to affect longevity in yeast, worms, fruit flies, and mice [3–6].

Another category of genes implicated in longevity modulation encode regulatory proteins, such as transcription factors and histone deacetylases (HDACs). HDACs remove acetyl groups from the lysine residues within histones and a range of other proteins [7]. Nuclear HDACs regulate transcription by modifying chromatin structure and transcription factors. There are Zn-dependent HDACs (classes I, II, and IV) and NAD-dependent HDACs (class III, the sirtuins) [7]. Genetic studies have implicated two nuclear HDACs in longevity extension of *Drosophila*, dRPD3 (class I) [8] and dSIR2 (class III) [9]. While the complete removal of dRPD3 is lethal [10], flies heterozygous for *rpd3* mutations have extended longevity [8]. Removal of dSIR2 is not lethal and has no longevity phenotype by itself, but overexpression of dSIR2 extends life span [9]. As with dRPD3, dosage appears to be crucial in that moderate global overexpression mediates life span increases but more pronounced overexpression is deleterious [11, 12]. Recently it was shown that knockdown of two class I HDACs in *C. elegans* increases life span, but worms null for these gene products have reduced life spans [13]. Several groups have shown that inhibitors of Zn-dependent HDACs such as suberoyanilide hydroxamic acid (SAHA) extend fly longevity [14–16]. Another inhibitor of Zn-dependent HDACs, beta-hydroxybutyrate, has been shown to extend *C. elegans* longevity [13]. In addition, removal of RPD3 in *S. cerevisae* leads to longevity extension [17, 18].

The capacity of single gene mutations to extend longevity was preceded by the discovery that nutrient uptake can affect both longevity and aging. Dietary restriction (DR) was first discovered in rodents, but has been shown to affect longevity in a variety of vertebrate and invertebrate species [19–21]. DR is defined as a level of nutrient intake that is reduced relative to *ad libitum* feeding without malnutrition. While this often means a restricted feeding schedule for rodents, for invertebrates other means need to be employed to restrict food uptake. DR in *Drosophila* can be achieved by formulating food of different nutrient levels using one of three general options: variation of carbohydrate, protein, or total nutrient (protein and carbohydrate) content [22–24]. Standard fly diets are usually optimized for reproduction (egg laying and larval growth), whereas peak DR-mediated longevity extension involves less nutrients than the standard diet. For the purposes of DR studies the standard diet can simply be regarded as a starting point for the manipulation of nutrient levels.

Our previous study on *rpd3*-mediated longevity changes in *Drosophila melanogaster* [8] used two nutrient conditions, standard (corn) and reduced (0.5N). However, a full range of nutrient levels is necessary to detect potential changes in life span relative to standard nutrients [23, 24, 26]. A report that *Drosophila rpd3* mRNA level is affected by nutrients [27] lends further impetus to a more thorough examination of the interplay between dRPD3, nutrition, and longevity. In the studies presented here, we determined if there is *rpd3*-mediated longevity extension at different nutrient levels.

## RESULTS

### Nutrients and RPD3 have additive effects upon longevity

This study utilizes the same alleles as our previous report [8], *rpd3^def^* (null), *rpd3^P-UTR^* (hypomorphic), and *rpd3^P-1.8^* (control), and varies complete nutrients (both sucrose and yeast) from 0.3N to 1.5N relative to standard 1.0N conditions. The *rpd3^def^* and *rpd3^P-UTR^* alleles are lethal when homozygous [10]. In all survival experiments using the *rpd3^def^* allele, including the study of flies that are double mutants for *rpd3* and *Thor*, crosses prior to the life span measurements yielded flies with matched genetic backgrounds (a hybrid mix of the *rpd3* stock and the *yw* strain, the latter being wild type for all genes related to this study). *rpd3^P-UTR^* has greatly reduced transcription in all tissues, whereas *rpd3^P-1.8^* only has reduced transcription in the eye [10, 28]. All survival experiments using the *rpd3^P-UTR^* and *rpd3^P-1.8^* alleles also had matched genetic backgrounds, derived from crosses to *CS* flies. Heterozygous male flies (20 days old) carrying the *rpd3^def^* and *rpd3^P-UTR^* alleles have 40% and 25% reduced levels, respectively, of *rpd3* mRNA (Supplementary Figure 1).

**Figure 1:**
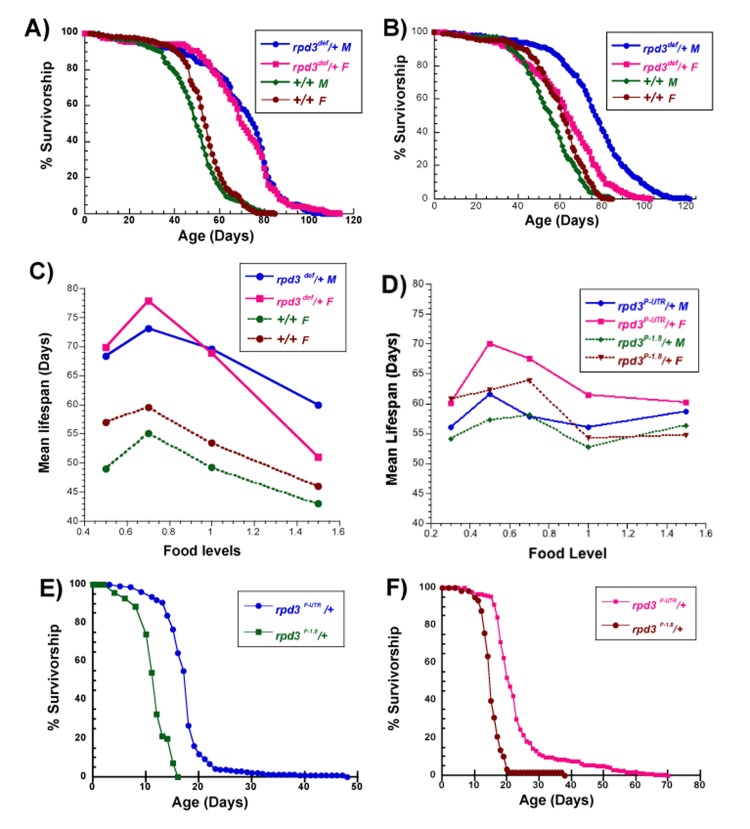
Longevity of rpd3 heterozygous mutant flies (**A**) Survivorship curves of male and female *rpd3^def^/+* and +/+ control flies on 1.0N (**A**) and corn food (**B**). Male *rpd3^def^/+* flies have increased mean and maximal longevity on both food levels compared to controls. Female *rpd3^def^/+* flies have maximal longevity increased on both food levels but mean is increased on 1.0N. (**C**) Mean longevity of *rpd3^def^/+* and *+/+* control flies, male and female, on food levels ranging from 0.5N to 1.5N. (**D**) Mean longevity of *rpd3^P-UTR^/+* and *rpd3^P-1.8^/+* control flies, male and female, on food levels ranging from 0.3N to 1.5N. **(E**, **F**) Survivorship of male (**E**) and female (**F**) *rpd3^P-UTR^/+* and *rpd3^P-1.8^/+* controls, on 0.1N food. See Tables 1–4 for number of flies in each experiment and statistical analysis.

Long-lived *rpd3* mutant flies exhibited nutrient-mediated changes in longevity. While a diet containing corn meal and yeast is often considered standard (hereafter called corn), the standard food used in this study contained equivalent calories but was formulated using sucrose and yeast, called 1.0N [26, 29]. Males heterozygous for *rpd3^def^* have extensions of 37% and 42% in mean longevity on corn and 1.0N food, respectively, compared to genetic controls (Figure 1A, B). Female flies heterozygous for the same allele have 4% and 31% longevity extension on corn and 1.0N food (Figure 1A, B, Table 1, 2). Mean life spans at different nutrient levels are shown for heterozygotes carrying the *rpd3^def^* allele in Figure 1C and heterozygotes carrying the *rpd3^P-UTR^* and *rpd3^P-1.8^* alleles in Figure 1D (Table 1, 2). Male and female flies carrying the *rpd3^def^* allele have extended longevity at all nutrient levels compared to genetic controls, while also showing increases and decreases in mean life span mediated by nutrients. Life span extensions for males vary between 36-41% compared to controls at the same food levels, while for females they vary between 12% at 1.5 food and 33% at 0.7N food. Male and female flies carrying the second allele, *rpd3^P-UTR^*, have smaller but consistent extensions in life span compared to flies carrying the control *rpd3^P-1.8^* allele (at most food levels). Females have 10-13% extensions at 0.5N, 1.0N, and 1.5N food levels, whereas males have extensions of 6-7% at 0.3N, 0.5N and 1.0N food levels.

**Table 1:** Effect of *rpd3^def^* on longevity of flies on different food levels

Gender	Genotype	Food (N)	Number	Mean Lifespan (% Change to controls)	X^2^	*p*	Maximal Lifespan (% Change)
M	+/+	1.0	261	49.7			71.3 (32)
M	*rpd3^def^/+*	1.0	236	70.6 (42)	250.01	<0.001*	93.5 (31)
M	+/+	0.5	261	50.4			64.8
M	*rpd3^def^/+*	0.5	247	68.8 (37)	283.64	<0.001*	95.8 (48%)
M	+/+	0.7	263	55.4			73.1
M	*rpd3^def^/+*	0.7	243	75.2 (36)	254.15	<0.001*	103.6 (42)
M	+/+	1.5	244	44.4			65
M	*rpd3^def^/+*	1.5	254	61.2 (39)	205.28	<0.001*	84 (30)
M	+/+	Corn	263	55.7			76
M	*rpd3^def^/+*	Corn	240	76.5 (37)	243.83	<0.001*	105.3 (39)
F	+/+	1.0	272	54.1			72.6
F	*rpd3^def^/+*	1.0	269	70.8 (31)	241.92	<0.001*	95.5 (32)
F	+/+	0.5	264	57.6			72.9
F	*rpd3^def^/+*	0.5	259	70.4 (22)	230.55	<0.001*	84.1 (15)
F	+/+	0.7	275	59.8			76.7
F	*rpd3^def^/+*	0.7	251	79.6 (33)	317.80	<0.001*	102.8 (34)
F	+/+	1.5	275	46.1			63
F	*rpd3^def^/+*	1.5	278	51.5 (12)	37.68	<0.001*	71 (12)
F	+/+	Corn	257	60.2			78
F	*rpd3^def^/+*	Corn	241	62.8 (4)	24.68	<0.001*	88.8 (14)

The mean and maximal lifespan of male (M) and female (F) *rpd3^def^/+* and +/+ control flies on different food levels. All values obtained for controls are compared to values of the *rpd3^def^/+* group of the same sex and on the same food levels to determine the percent increase in mean and maximal lifespan. Food (N): standard food is 1.0N. Number: number of flies used in the experiment. Mean and maximal lifespan are in days. Data are censored for 0-10 days. Long-rank analyses were performed using the JMP 11 program.

**Table 2:** Effect of DR on longevity of *rpd3^def^/+* (Top) and +/+ control (Bottom) flies

Gender	Genotype	Food (N)	Number	Mean Lifespan (% Change to controls)	X^2^	*p*	Maximal Lifespan (% Change)
M	*rpd3^def^/+*	1.0	236	70.6			93.5
M	*rpd3^def^/+*	0.5	247	68.8 (−3)	3.6229	0.57	95.8 (2)
M	*rpd3^def^/+*	0.7	243	75.2 (7)	16.91	<0.001*	103.6 (11)
M	*rpd3^def^/+*	1.5	254	61.2 (−13)	58.619	<0.001*	84 (−10)
M	*rpd3^def^/+*	Corn	240	76.5 (8)	21.005	<0.001*	105.3 (13)
F	*rpd3^def^/+*	1.0	269	70.8			95.5
F	*rpd3^def^/+*	0.5	259	70.4 (0)	4.6814	0.0305*	84.1 (−12)
F	*rpd3^def^/+*	0.7	251	79.6 (12)	40.489	<0.001*	102.8 (8)
F	*rpd3^def^/+*	1.5	278	51.5 (−27)	266.83	<0.001*	71 (−26)
F	*rpd3^def^/+*	Corn	241	62.8 (−11)	21.061	<0.001*	88.8 (7)
M	+/+	1.0	261	49.7			71.3
M	+/+	0.5	261	50.4 (1)	0.3308	0.5652	64.8 (−9)
M	+/+	0.7	263	55.4 (11)	21.498	<0.001*	73.1 (3)
M	+/+	1.5	244	44.4 (−11)	24.249	<0.001*	65 (−9)
M	+/+	Corn	263	55.7 (12)	25.086	<0.001*	76 (7)
F	+/+	1.0	272	54.1			72.6
F	+/+	0.5	264	57.6 (6)	17.045	<0.001*	72.9 (0)
F	+/+	0.7	275	59.8 (10)	44.583	<0.001*	76.7 (6)
F	+/+	1.5	275	46.1 (−15)	62.478	<0.001*	63 (−13)
F	+/+	Corn	257	60.2 (11)	52.928	<0.001*	78 (7)

The mean and maximal lifespan of male (M) and female (F) *rpd3^def^/+* (Top) and +/+ control (Bottom) flies on different food levels. All values are compared to values obtained for the flies of the same sex and of the same genotype kept on 1.0N food to determine the percent increase in mean and maximal lifespan. Food N: Number: number of flies used in the experiment. Mean and maximal lifespan are in days. Data are censored for 0-10 days. Long-rank analyses were performed using the JMP 11 program.

Some nutrient-mediated changes in life span differed for flies carrying the two *rpd3* alleles. In male and female flies carrying the *rpd3^def^* allele and control flies for both alleles (*rpd3^def^* controls, and *rpd3^P-1.8^*) the peak extension due to DR (maximal extension of mean longevity compared to 1.0N food) occurs at 0.7N (Figure 1C,D and Table 1–4). However, in male and female flies carrying the *rpd3^P-UTR^* allele the peak extension due to DR occurs at 0.5N (Figure 1D, and Table 3, 4). The effect of high calories (1.5N) also differed. The mean life span is unchanged for *rpd3^P-UTR^* and *rpd3^P-1.8^* flies on 1.5N and 1.0N food, but for *rpd3^def^* and *rpd3^def^* controls the mean life span is lower on 1.5N compared to 1.0N food. Since all of the survival studies reported here were performed at the same time with the same batches of food, precluding food preparation and fly culture as sources of variability, and since the two controls for each allele exhibited different responses to high calorie food (1.5N), it is likely that the response to high calorie food was affected by the genetic background. It has been shown by other groups that flies with different genetic backgrounds can differ in their responses to nutrients [30].

**Table 3:** Effect of *rpd3^P-UTR^/+* mutation on longevity of flies on different food levels

Gender	Genotype	Food (N)	Number	Mean Lifespan (% Change to controls)	X^2^	*p*	Maximal Lifespan (% Change)
M	*rpd3^P-1.8^/+*	1.0	228	54.2			70.9
M	*rpd3^P-UTR^/+*	1.0	219	57.4 (6)	10.212	0.0014	72.61 (2)
M	*rpd3^P-1.8^/+*	0.1	70	11.6			15.4
M	*rpd3^P-UTR^/+*	0.1	237	17.6 (51)	206.86	<0.001*	26.1 (70)
M	*rpd3^P-1.8^/+*	0.3	226	54.5			66.6
M	*rpd3^P-UTR^/+*	0.3	206	58.1 (7)	36.901	<0.001*	71.1 (7)
M	*rpd3^P-1.8^/+*	0.5	223	58.7			74.4
M	*rpd3^P-UTR^/+*	0.5	225	62.4 (7)	23.307	<0.001*	85.7 (15)
M	*rpd3^P-1.8^/+*	0.7	227	60.3			75.6
M	*rpd3^P-UTR^/+*	0.7	213	58.7 (−3)	5.7376	0.0166*	74.6 3 (−1)
M	*rpd3^P-1.8^/+*	1.5	228	58.0			73.5
M	*rpd3^P-UTR^/+*	1.5	231	60.0 (3)	4.4888	0.0341*	76.8 (5)
M	*rpd3^P-1.8^/+*	Corn	227	65.5			87.8
M	*rpd3^P-UTR^/+*	Corn	218	68.1 (1)	0.0645	0.7995	82.6 (−6)
F	*rpd3^P-1.8^/+*	1.0	216	56.2			69.9
F	*rpd3^P-UTR^/+*	1.0	223	63.0 (12)	83.590	<0.001*	79.4 (24)
F	*rpd3^P-1.8^/+*	0.1	58	15.6			23.2
F	*rpd3^P-UTR^/+*	0.1	219	23.6 (52)	120.64	<0.001*	48.7 (110)
F	*rpd3^P-1.8^/+*	0.3	225	61.0			78
F	*rpd3^P-UTR^/+*	0.3	199	61.9 (2)	9.0925	0.0026	80.7 (4)
F	*rpd3^P-1.8^/+*	0.5	224	64.0			77.5
F	*rpd3^P-UTR^/+*	0.5	218	72.4 (13)	112.55	<0.001*	89.2 (15)
F	*rpd3^P-1.8^/+*	0.7	224	67.3			80.8
F	*rpd3^P-UTR^/+*	0.7	232	68.8 (2)	13.142	0.0003*	85.5 ()6
F	*rpd3^P-1.8^/+*	1.5	230	56.6			71.4
F	*rpd3^P-UTR^/+*	1.5	226	63.0 (10)	60.616	<0.001*	80.6 (13)
F	*rpd3^P-1.8^/+*	Corn	224	55.0			76.1
F	*rpd3^P-UTR^/+*	Corn	230	57.0 (4)	24.759	<0.001*	88.8 (17)

The mean and maximal lifespan of male (M) and female (F) *rpd3^P-UTR^/+* and *rpd3^P-1.8^/+* control flies on different food levels. All values for control group are compared to values of *rpd3^P-UTR^/+* flies of the same sex and on the same food levels to determine the percent increase in mean and maximal lifespan. Food (N): standard food is 1.0N. Number: number of flies used in the experiment. Mean and maximal lifespan are in days. Data are censored for 0-10 days except for longevity experiments on 0.1N. Long-rank analyses were performed using the JMP 11 program.

**Table 4:** Effects of DR on mean and maximal longevity of *rpd3^P-UTR^/+* (Top) and *rpd3^P-1.8^/+* (Bottom) flies

Gender	Genotype	Food (N)	Number	Mean Lifespan (% Change to controls)	X^2^	*p*	Maximal Lifespan (% Change)
M	*rpd3^P-UTR^/+*	1.0	219	57.4			72.61
M	*rpd3^P-UTR^/+*	0.1	237	17.6 (−70)	506.18	<0.001*	26.1(−64)
M	*rpd3^P-UTR^/+*	0.3	206	58.1 (1)	0.0011	0.9732	71.1 (−2)
M	*rpd3^P-UTR^/+*	0.5	225	62.4 (8)	31.336	<0.001*	85.7 (18)
M	*rpd3^P-UTR^/+*	0.7	213	58.7 (2)	6.6398	0.0100*	74.6 (3)
M	*rpd3^P-UTR^/+*	1.5	231	60.0 (4)	8.9391	0.0028	76.8 (6)
M	*rpd3^P-UTR^/+*	Corn	218	68.1 (19)	129.48	<0.001*	82.6 (14)
F	*rpd3^P-UTR^/+*	1.0	223	63.0			79.4
F	*rpd3^P-UTR^/+*	0.1	219	23.6 (−62)	423.72	<0.001*	48.7 (−39)
F	*rpd3^P-UTR^/+*	0.3	199	61.9 (−2)	0.7415	0.3892	80.7 (2)
F	*rpd3^P-UTR^/+*	0.5	218	72.4 (15)	90.451	<0.001*	89.2 (12)
F	*rpd3^P-UTR^/+*	0.7	232	68.8 (9)	52.246	<0.001*	85.5 (8)
F	*rpd3^P-UTR^/+*	1.5	226	63.0 (0)	1.4257	0.2325	80.6 (2)
F	*rpd3^P-UTR^/+*	Corn	230	57.0 (−10)	2.8411	0.0919	88.8 (12)
M	*rpd3^P-1.8^/+*	1.0	228	54.2			70.9
M	*rpd3^P-1.8^/+*	0.1	70	11.6 (−79)	437.4	<0.001*	15.4 (−78)
M	*rpd3^P-1.8^/+*	0.3	226	54.5 (0)	4.0704	0.0436*	66.6 (−6)
M	*rpd3^P-1.8^/+*	0.5	223	58.7 (8)	19.005	<0.001*	74.4 (5)
M	*rpd3^P-1.8^/+*	0.7	227	60.3 (11)	53.962	<0.001*	75.6 (7)
M	*rpd3^P-1.8^/+*	1.5	228	58.0 (7)	19.156	<0.001*	73.5 (4)
M	*rpd3^P-1.8^/+*	Corn	227	65.5 (21)	126.69	<0.001*	87.8 (24)
F	*rpd3^P-1.8^/+*	1.0	216	56.2			69.9
F	*rpd3^P-1.8^/+*	0.1	58	15.6 (−72)	398.44	<0.001*	23.2 (−67)
F	*rpd3^P-1.8^/+*	0.3	225	61.0 (8)	26.974	<0.001*	78 (12)
F	*rpd3^P-1.8^/+*	0.5	224	64.0 (14)	80.351	<0.001*	77.5 (11)
F	*rpd3^P-1.8^/+*	0.7	224	67.3 (20)	163.90	<0.001*	80.8 (16)
F	*rpd3^P-1.8^/+*	1.5	230	56.6 (1)	0.3637	0.5465	71.4 (2)
F	*rpd3^P-1.8^/+*	Corn	224	55.0 (−2)	1.2871	0.2566	76.1 (9)

The mean and maximal lifespan of male (M) and female (F) *rpd3^P-UTR^/+* (Top) and *rpd3^P-1.8^/+* (Bottom) control flies on different food levels. All values are compared to values obtained for flies of the same sex and the same genotype kept on 1.0N food to determine the percent increase in mean and maximal lifespan. Food (N): standard food is 1.0N. Number: number of flies used in the experiment. Mean and maximal lifespan are in days. Data are censored for 0-10 days. Long-rank analyses were performed using the JMP 11 program.

It is of interest that there is a DR peak for flies carrying both the null and hypomorphic *rpd3* alleles, despite the potentially confounding effects of the two hybrid genetic backgrounds used in these experiments. For males carrying either allele, the DR peak is 7-9% higher than the mean life span at 1.0N, whereas for females carrying either allele the DR peak is 12-15% higher. Male controls for both alleles have a DR peak of 11%, whereas the female controls for *rpd3^def^* have a DR peak of 11% and the female controls for *rpd3^P-UTR^* have a DR peak of 20%. Finally, there is a trend when comparing mutants to controls. For both males and females carrying the *rpd3^P-UTR^* allele, the DR peak is lower in mutants compared to controls. A similar trend is seen for males but not females carrying the *rpd3^def^* allele.

### *rpd3* mutants live longer under conditions close to starvation

Decreasing food below the level that yields DR induces increasing starvation. Flies raised on 0.1N food since eclosion have much shorter life spans compared to flies raised on all higher nutrient levels, indicating a condition close to starvation. *rpd3^P-UTR^* flies live longer on 0.1N food compared to controls, indicating increased resistance to severe food deprivation (Figure 1E,F).

### Effects of *rpd3* on the expression of 4E-BP and Tor

**Figure 2:**
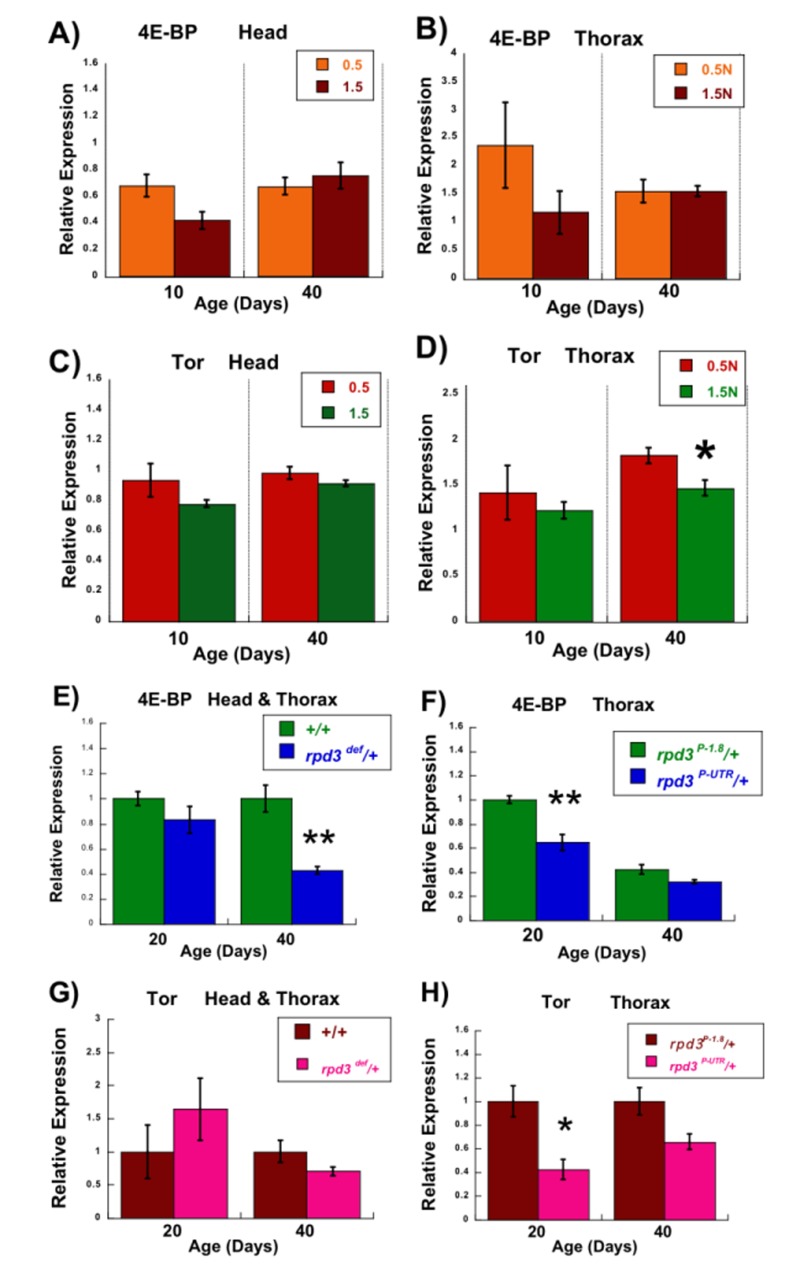
Transcription of the 4E-BP and Tor genes under conditions extending longevity. (A-D) Effects of DR on levels of 4E-BP and Tor mRNA Average expression of mRNA for the *4E-BP* (**A, B**) and *Tor* (**C**, **D**) genes in three biological replicates isolated from the heads (**A, C**) and thoraces (**B, D**) of wild type *CS* female flies kept on 0.5N and 1.5N food at 10 or 40 days of age by Q-PCR. *p<0.05, 25 heads or thoraces per replicate. (**E- H**) **Effects of *rpd3* mutations on the levels of *4E-BP* and *Tor* mRNA** (**E**) Average expression of mRNA for the *4E-BP* gene isolated from the heads and thoraces of *rpd3^def^/+* and +/+ control male flies at 20 and 40 days of age by Q-PCR. (**F**) Average expression of mRNA for the *4E-BP* gene isolated from thoraces of *rpd3^P-UTR^/+* and control *rpd3^P-1.8^/+* male flies at 20 or 40 days of age by Q-PCR. (**G**) Average expression of mRNA for the *Tor* gene isolated from the heads and thoraces of *rpd3^def^/+* and +/+ control male flies at 20 and 40 days of age by Q-PCR. (**H**) Average expression of mRNA for the *Tor* gene isolated from the thoraces of *rpd3^P-UTR^/+* and control *rpd3^P-1.8^/+* male flies at 20 and 40 days of age by Q-PCR. In each case three biological replicates were used. Graphs are plotted as the means +/− standard errors. *p<0.05 **p<0.01, 25 heads + thoraces or thoraces per replicate.

Our results indicate that decreased *rpd3* mRNA levels has a minor effect on DR-mediated longevity extension and a major effect on resistance to food deprivation. In order to gain more insight into the interaction between RPD3 and nutrient responses, we determined whether as a transcriptional regulator RPD3 might be affecting the mRNA levels for two post-transcriptional modulators of nutrient signaling, 4E-BP and Tor (Figure 2).

4E-BP regulates mRNA translation and the Tor kinase has a central role in nutrient-mediated signal transduction [31]. We first determined whether *4E-BP* and *Tor* mRNA levels are affected by nutrients in wild type *Drosophila*. Heads and thoraces were assayed separately, in case changes were localized to one area of the body. Young and old flies were also assayed, in case changes were affected by aging. Young (10 day) wild type female flies have modestly increased *4E-BP* mRNA levels at 0.5N compared to 1.5N (similar trends in head and thorax, though the changes are not signifi-cant), whereas older (40 day) flies show no changes (Figure 2A,B). Another group has shown that 4E-BP protein levels are increased under DR conditions [32].

*Tor* mRNA shows much smaller changes, comparing 0.5N and 1.5N of young and old flies (Figure 2C,D), though the change in 40 day thoraces is significant. It was then determined whether *rpd3* mutations affect the transcription of *4E-BP* and *Tor*. When heterozygotes for the *rpd3^def^* allele are compared to genetic controls, young mutant male flies (20 days) show no changes in *4E-BP* mRNA levels but older mutant flies (40 days) have 60% less mRNA (Figure 2E). Male flies heterozygous for the *rpd3^P-UTR^* allele have a 40% reduction in *4E-BP* mRNA levels when younger and a smaller decrease relative to controls when older (only thoraces were assayed, see Methods) (Figure 2F). Flies heterozygous for the *rpd3^def^* allele have no significant changes in *Tor* mRNA level at both ages compared to genetic controls (Figure 2G). Flies heterozygous for the *rpd3^P-UTR^* allele have a 60% reduction in *Tor* mRNA when young and tend towards a decrease relative to controls when older (though this is not significant) (Figure 2H). In summary, the transcription of *4E-BP* is affected by both *rpd3* alleles, whereas only one allele affects *Tor* transcription. In all cases where there were significant changes, reduced dRPD3 levels led to decreased transcription. DR and *rpd3* mutants differed in their effects upon *4E-BP* and *Tor* transcription, providing further evidence that dRPD3 and nutrient status differ in their mechanism of life span extension.

### RPD3 and 4E-BP have distinct effects on longevity

**Figure 3:**
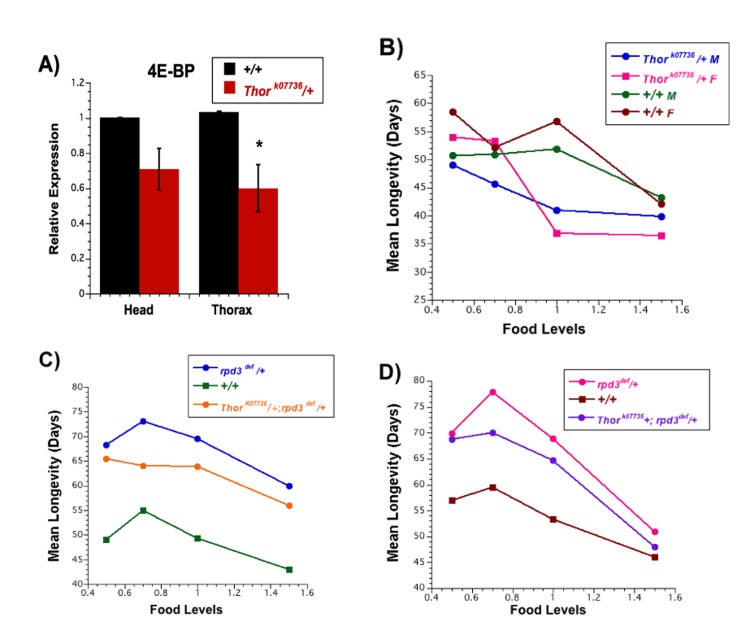
Longevity of flies carrying mutations in both rpd3 and Thor (4E-BP) (**A**) Average expression of mRNA for the *4E-BP* gene in two (*yw*) or three (*Thor* mutant) biological replicates isolated from the heads and thoraces of female flies at 10 days of age by Q-PCR. Black, *+/+* controls, magenta, *Thor^k07736^/+*. Graphs are plotted as the means +/− standard errors. (**B**) Mean life span of *Thor^k0773^/+* and *+/+* control (*yw* background) flies, male and female, on 0.5N, 0.7N, 1.0N, and 1.5N food levels.(**C**, **D**) Mean life span of male (**C**) and female (**D**) *rpd3^def^/+, Thor^k0773^* /+; *rpd3^def^*/+ double mutants and +/+ control flies on 0.5N, 0.7N, 1.0N, and 1.5 food levels.

Several groups have reported that flies lacking any 4E-BP have lowered life spans at all nutrient levels [24, 32–34]. Since *rpd3-*mutant flies have moderately reduced levels of *4E-BP* mRNA, we asked whether a moderate reduction in *4E-BP* mRNA might affect life span. This was measured by using a hypomorphic allele of the *4E-BP* gene, *Thor^k07736^*. Flies carrying this allele were backcrossed to *yw* flies for 10 generations. Hetero-zygotes carrying the backcrossed *Thor^k07736^* (4E-BP) allele have a 30% reduction in *4E-BP* mRNA (Figure 3A). Heterozygous *Thor* males have mean life spans that are reduced by 19%, 7%, and 5% relative to *yw* controls at 1.0N, 0.7N, and 0.5N food levels, respectively (no change at 1.5N) (Figure 3B, Table 5, 6). Heterozygous *Thor* females have mean life spans that are reduced by 12%, 33%, and 9% relative to *yw* controls at 1.5N, 1.0N, and 0.5N (no change at 0.7N) (Figure 3B, Table 5, 6). There were several additional differences between *Thor* mutants and their controls. Over a food range of 0.5N to 1.5N, *yw* controls show a peak life span at 1.0N, whereas *Thor* mutants have a peak at 0.5N (Figure 3B, Table, 5, 6). In addition, 1.5N food decreased the mean life spans of *yw* controls relative to 1.0N food but not the mean life spans of the *Thor* mutants. These differences between *Thor* mutants and the controls provide some evidence that 4E-BP can affect the response to nutrients.

**Table 5:** Effect of *4E-BP* mutation on longevity of flies on different food levels

Gender	Genotype	Food (N)	Number	Mean Lifespan (% Change to controls)	X^2^	*p*	Maximal Lifespan (% Change)
M	*+/+*	1.0	273	52.7			73.8
M	*Thor^k07736^/+*	1.0	209	42.8 (−19)	55.70	<0.001*	66.1 (−10)
M	*+/+*	0.5	286	51.7			72.6
M	*Thor^k07736^/+*	0.5	231	49.2 (−5)	5.62	0.0178*	66 (−8)
M	*+/+*	0.7	277	51.1			73.5
M	*Thor^k07736^/+*	0.7	221	47.6 (−7)	9.68	0.0019*	69.8 (−5)
M	*+/+*	1.5	277	43.3			64.93
M	*Thor^k07736^/+*	1.5	201	41.8 (−3)	3.38	0.0658	58.3 (−10)
M	*+/+*	Corn	280	54.0			71.8
M	*Thor^k07736^/+*	Corn	220	42.8 (1)	3.58	0.0583	79.0 (10)
F	*+/+*	1.0	273	57.6			74.2
F	*Thor^k07736^/+*	1.0	226	38.3 (−33)	317.08	<0.001*	56.0 (−25)
F	*+/+*	0.5	247	59.9			78.9
F	*Thor^k07736^/+*	0.5	231	54.6 (−9)	22.99	<0.001*	72 (−8)
F	*+/+*	0.7	282	52.5			71
F	*Thor^k07736^/+*	0.7	234	54.3 (3)	12.69	0.0004*	74.5 (5)
F	*+/+*	1.5	284	42.7			59.6
F	*Thor^k07736^/+*	1.5	220	37.5 (−12)	36.98	<0.001*	52.9 (−11)
F	*+/+*	Corn	273	58.6			75.9
F	*Thor^k07736^/+*	Corn	222	59.4 (1)	4.26	0.039	78.0 (3)

The mean and maximal lifespan of male (M) and female (F) +/+ control and *4E-BP (Thor^k07736^/+)* mutant flies on different food levels. All *+/+* values are compared to values of *Thor^k07736^/+* group on the same food level and same sex to determine the percent increase in mean and maximal lifespan in 4E-BP mutant flies. Food (N): standard food is 1.0N. Number: number of flies used in the experiment. Mean and maximal lifespan are in days. Data are censored for 0-10 days. Long-rank analyses were performed using the JMP 11 program.

**Table 6:** Effect of DR on longevity of *4E-BP* hypomorphic (Top) and control (Bottom) flies

Gender	Genotype	Food (N)	Number	Mean Lifespan (% Change to controls)	X^2^	*p*	Maximal Lifespan (% Change)
M	*Thor^k07736^/+*	1.0	209	42.8			66.1
M	*Thor^k07736^/+*	0.5	231	49.2 ()	16.914	<0.001*	66 (0)
M	*Thor^k07736^/+*	0.7	221	47.6 ()	12.807	0.0003*	69.8 (6)
M	*Thor^k07736^/+*	1.5	201	41.8 ()	0.7227	0.3953	58.3 (−12)
M	*Thor^k07736^/+*	corn	220	42.8 ()	80.044	<0.001*	79.0 (20)
F	*Thor^k07736^/+*	1.0	226	38.3 ()			56.0
F	*Thor^k07736^/+*	0.5	231	54.6 ()	208.70	<0.001*	72 (29)
F	*Thor^k07736^/+*	0.7	234	54.3 ()	191.31	<0.001*	74.5 (33)
F	*Thor^k07736^/+*	1.5	220	37.5 ()	0.2207	0.6385	52.9 (6)
F	*Thor^k07736^/+*	corn	222	59.4 ()	282.08	<0.001*	78.0 (40)
M	*+/+*	1.0	273	52.7			73.8
M	*+/+*	0.5	286	51.7	1.745	0.1865	72.6 (2)
M	*+/+*	0.7	277	51.1	0.5569	0.4555	73.5 (0)
M	*+/+*	1.5	277	43.3	58.062	<0.001*	64.93 (−12)
M	*+/+*	corn	280	54.0	2.1704	0.1407	71.8 (−3)
F	*+/+*	1.0	273	57.6			74.2
F	*+/+*	0.5	247	59.9	10.077	0.0015*	78.9 (6)
F	*+/+*	0.7	282	52.5	28.028	<0.001*	71 9 (−4)
F	*+/+*	1.5	284	42.7	226.87	<0.001*	59.6 (−20)
F	*+/+*	corn	273	58.6	2.81	0.0937	75.9 (2)

The mean and maximal lifespan of male (M) and female (F) *4E-BP (Thor^k07736^/+)* (Top) mutant and control (Bottom) *+/+* flies on different food levels. All values are compared to values of flies the same sex and genotype kept on 1.0N food to determine the percent increase in mean and maximal lifespan. Food (N): standard food is 1.0N. Number: number of flies used in the experiment. Mean and maximal lifespan are in days. Data are censored for 0-10 days. Long-rank analyses were performed using the JMP 11 program.

We next examined the longevity of flies heterozygous for both *Thor^k07736^ (4E-BP)* and *rpd3^def^* to directly assess the combined effects of these gene products (Figure 3C,D, Table 7, 8). Preliminary experiments indicated that *rpd3* mutations mediated a decrease in *4E-BP* mRNA when the flies also carried a *Thor* mutation (double heterozygotes) (Fig. Supplemental 2), similar to the decrease mediated by *rpd3* mutations in the absence of *Thor* (Fig. 2). Double heterozygotes, single hetero-zygotes, and control flies shared the same genetic background. *rpd3*-mediated life span extension was attenuated but not eliminated by the presence of the *Thor* allele (Figure 3 C,D, Table, 7, 8). The reduction in mean life span of the double mutants relative to flies singly mutant for *rpd3^def^* ranged from 4% - 14% for males and 3% - 12% for females. While female flies that were doubly heterozygous maintained a smaller DR peak of 0.7N, male flies that were doubly heterozygous had no DR-mediated increase in life span. Doubly heterozygous flies of both sexes had decreased life spans at 1.5N relative to 1.0N. The simplest interpretation would be an overall superimposition of the life span decrease mediated by reduced 4E-BP on the life span extension mediated by reduced dRPD3.

**Table 7:** *4E-BP* (*Thor^k07736^*) mutation affects longevity of *rpd3^def^/+* flies on different food levels

Gender	Genotype	Food (N)	Number	Mean Lifespan (% Change to controls)	X^2^	*p*	Maximal Lifespan (% Change)
M	*rpd3^def^/+*	1.0	236	70.6			93.5
M	*Thor^k07736^/+;rpd3^def^/+*	1.0	256	64.3 (−9)	142.91	<0.001*	88.8 (−5)
M	*rpd3^def^/+*	0.5	247	68.8	3.6229	0.57	95.8
M	*Thor^k07736^/+;rpd3^def^/+*	0.5	259	65.9 (−4)	237.93	<0.001*	87.4 (−9)
M	*rpd3^def^/+*	0.7	243	75.2	16.912	<0.001*	103.6
M	*Thor^k07736^/+;rpd3^def^/+*	0.7	244	64.7 (−14)	101.36	<0.001*	87.0 (−16)
M	*rpd3^def^/+*	1.5	254	61.2	58.619	<0.001*	84
M	*Thor^k07736^/+;rpd3^def^/+*	1.5	228	57.1 (−7)	118.91	<0.001*	75.1 (11)
M	*rpd3^def^/+*	Corn	240	76.5	21.005	<0.001*	105.3
M	*Thor^k07736^/+;rpd3^def^/+*	Corn	242	68.5 (10)	124.82	<0.001*	90.9 (14)
F	*rpd3^def^/+*	1.0	269	70.8			95.5
F	*Thor^k07736^/+;rpd3^def^/+*	1.0	276	64.8 (−8)	130.99	<0.001*	89.0 (−7)
F	*rpd3^def^/+*	0.5	259	70.4	4.6814	0.0305*	84.1
F	*Thor^k07736^/+;rpd3^def^/+*	0.5	274	68.6 (−3)	164.07	<0.001*	87.9 (5)
F	*rpd3^def^/+*	0.7	251	79.6	40.489	<0.001*	102.8
F	*Thor^k07736^/+;rpd3^def^/+*	0.7	275	69.9 (−12)	105.99	<0.001*	94.5 (−8)
F	*rpd3^def^/+*	1.5	278	51.5	266.83	<0.001*	71
F	*Thor^k07736^/+;rpd3^def^/+*	1.5	263	49.5 (−4)	16.049	<0.001*	70.1 (0)
F	*rpd3^def^/+*	Corn	241	62.8	21.061	<0.001*	88.8
F	*Thor^k07736^/+;rpd3^def^/+*	Corn	258	62.2 (−1)	5.401	0.0201*	78 (−12)

The mean and maximal lifespan of male (M) and female (F) *rpd3^def^/+* and *Thor^k07736^/+;rpd3^def^/+* mutant flies on different food levels. All *rpd3^def^/+* values are compared to *Thor^k07736^/+;rpd3^def^/+* value mutant of the same sex and on the same food level to determine the percent increase in mean and maximal lifespan. Food (N): standard food is 1.0N. Number: number of flies used in the experiment. Mean and maximal lifespan are in days. Data are censored for 0-10 days. Long-rank analyses were performed using the JMP 11 program.

**Table 8:** *4E-BP* mutation affects longevity of *rpd3^def^/+* flies on different food levels

Gender	Genotype	Food (N)	Number	Mean Lifespan (% Change to controls)	X^2^	*p*	Maximal Lifespan (% Change)
M	*Thor^k07736^/+;rpd3^def^/+*	1.0	256	64.3			88.8
M	*Thor^k07736^/+;rpd3^def^/+*	0.5	259	65.9 (2)	2.1279	0.1446	87.4 (−2)
M	*Thor^k07736^/+;rpd3^def^/+*	0.7	244	64.7 (0)	1.34	0.247	87.0 (−2)
M	*Thor^k07736^/+;rpd3^def^/+*	1.5	228	57.1 (−11)	38.915	<0.001*	75.1 (−15)
M	*Thor^k07736^/+;rpd3^def^/+*	Corn	242	68.5 (6)	15.525	<0.001*	90.9 (2)
F	*Thor^k07736^/+;rpd3^def^/+*	1.0	276	64.8			89.0
F	*Thor^k07736^/+;rpd3^def^/+*	0.5	274	68.6 (6)	8.6198	0.0033*	87.9 (−1)
F	*Thor^k07736^/+;rpd3^def^/+*	0.7	275	69.9 (8)	11.562	0.0007*	94.5 (6)
F	*Thor^k07736^/+;rpd3^def^/+*	1.5	263	49.5 (−24)	165.63	<0.001*	70.1 (−21)
F	*Thor^k07736^/+;rpd3^def^/+*	Corn	258	62.2 (−4)	6.7179	0.0095*	78 (−12)

The mean and maximal lifespan of male (M) and female (F) *Thor^k07736^/+;rpd3^def^/+* mutant flies on different food levels. All values are compared to flies of the same sex kept on 1.0N food to determine the percent increase in mean and maximal lifespan. Food (N): standard food is 1.0N. Number: number of flies used in the experiment. Mean and maximal lifespan are in days. Data are censored for 0-10 days. Long-rank analyses were performed using the JMP 11 program.

In summary, DR-mediated longevity extension is maintained in the presence of *rpd3* mutations, though it may have a moderately reduced magnitude. Our epistasis experiments using flies doubly mutant for *rpd3* and *4E-BP* suggest potential crosstalk between dRPD3 and nutrient signaling.

## DISCUSSION

Many single gene mutations that extend life span in *Drosophila* implicate particular pathways in longevity regulation, such as insulin and Tor signaling [1]. Dietary restriction is thought to extend life span through nutrient signaling and multiple effector pathways (intermediary metabolism, flux through the mitochondrial respiratory chains, control of protein synthesis rates, to name a few). The complexities of nutrient signaling at the cellular and tissue level are paralleled by the complexities of measuring nutrient effects in survival studies, since many variables can affect nutrient effects upon life span. Our earlier study showed that flies heterozygous for mutations in *rpd3* extend longevity [8]. Connecting this transcriptional regulator to nutrient signaling would facilitate the identification of effectors for nutrient-mediated regula-tion, since some of the transcriptional targets of dRPD3 would be candidate effectors. The earlier study obtained preliminary evidence of a potential connection to DR. However, since only two nutrient levels were compared, corn and 0.5N, the present study aimed to more systematically test for a relationship between nutrient signaling and dRPD3-mediated longevity control. Our data indicate longevity extension by DR and dRPD3 are distinct, with some potential crosstalk or overlap.

Genetic background and gender have strong effects on longevity in *Drosophila*. The finding that reduced dRPD3 extends longevity has been shown using several genetic backgrounds and both sexes. In our earlier study, *rpd3^def^* flies were crossed to wild type *CS* flies prior to longevity measurements, whereas *rpd3^P-UTR^* and *rpd3^P-1.8^* were crossed to a *w^m4^* fly line (*rpd3^P-UTR^* had also been crossed into a *w^m4^* genetic background prior to the longevity studies). In the present study *rpd3^def^* flies were crossed to *yw* flies, whereas *rpd3^P-UTR^* and *rpd3^P-1.8^* flies were crossed to *CS* prior to longevity measurements (*yw* and *w^m4^* are control strains for any relevant genes). The amount of life span extension varies from males to females and also varies for each allele depending upon the genetic background. It is therefore noteworthy that longevity extension was consistently obtained under all conditions, and that this effect has been replicated by a different research group using a different genetic background [25]. The *Thor* (4E-BP) allele was backcrossed into a *yw* background. In the present study all experiments with the *rpd3^def^* allele involve crosses with flies containing a *yw* background, so that every longevity experiment using this allele (whether singly or in combination with the *Thor* allele) had the same background. This entails a 50%/50% mix of the genetic background from the *rpd3* mutant stocks (all *rpd3* alleles are from a P-element mutagenesis that used a *rosy* strain) and the *yw* stock.

Every longevity experiment using the *rpd3^P-UTR^* and *rpd3^P-1.8^* alleles has a 50%/50% mix of the *rpd3* stock and *CS* strain.

Longevity studies in budding yeast have shown that *rpd3* deletion extends replicative life span and *rpd3* deletion mutants raised on DR have no additional extension of life span [18]. A subsequent study implicated RPD3 in a pathway that can regulate life span independently of DR but that converges on a common effector, the yeast homolog of S6K, indicating a potential connection [35]. Ours is the first study in metazoans to examine the longevity effects of RPD3 on a range of nutrient levels. We aimed to engage all potential nutrient-sensing pathways by varying both sugar and protein (yeast).

Three types of nutrient effects were examined in this study: DR (0.5N and 0.7N food), severe nutrient deprivation (0.1N and 0.3N food), and nutrient excess (1.5N food). Earlier studies used only one nutrient condition for DR (0.5N) [8, 9]. We observed DR-mediated longevity extension with both *rpd3* alleles and both sexes. Decreased longevity on high calories (1.5N) relative to control food (1.0N) was only observed for the *rpd3^def^* allele (both sexes), though it was tested using both. The most severe nutrient deprivation (0.1N) was only tested using the *rpd3^P-UTR^* allele, with the mutant flies showing increased resistance to near starvation. This correlates with some but not all studies on the starvation resistance of DR flies. Such studies raise flies on standard or DR food, and then transfer the flies to conditions providing moisture but no nutrients. Virgin female flies subjected to DR were reported to have increased starvation resistance at younger ages but decreased resistance at later ages [36]. Young mated flies (both male and female) under DR were reported to have increased starvation resistance, but mated young male flies were also reported to have decreased resistance [37–39]. Genetic background, gender, and types of nutrients prior to starvation may account for some of the differences seen in starvation studies.

Since long-lived *rpd3* flies exhibited DR-mediated life span extension, it is likely that dRPD3 and DR operate by distinct mechanisms. However, our longevity data does not rule out an effect of DR upon dRPD3-mediated longevity regulation. Potential interactions could be either upstream or downstream of dRPD3. An example of upstream regulation would be evidence of nutrient effects on *rpd3* transcription. In one study newly eclosed flies were first fed a yeast-free diet (4 days) and then fed yeast (calorie and protein increase); addition of yeast led to an increase in *rpd3* transcription [27]. The same study examined the transcription profile of *Drosophila* S2 cells (embryonic origin) that express a constitutively active form of the transcription factor dFOXO. Since decreased insulin signaling and DR activate dFOXO, this serves as a surrogate for those conditions. Cells with activated dFOXO had a marked decrease in *rpd3* transcription. It is also possible that DR could have an effect downstream from dRPD3. For example, dRPD3 may modulate transcription of a gene that was also modulated by nutrients. We tested two genes that produce proteins representing convergence points for nutrient signaling, Tor and 4E-BP. Tor is a kinase that phosphorylates S6K and 4E-BP, thereby regulating metabolism and protein synthesis [31]. 4E-BP regulates translation in response to DR and other conditions [32]. Transcription of the *Drosophila* *4E-BP* gene is regulated by the dFOXO transcription factor in response to stress or starvation (40, 41]. 4E-BP protein levels are increased during DR in *Drosophila* and this increase has been linked to a variety of metabolic changes stimulated by DR [32]. We found that transcription of the *4E-BP* gene is also regulated by dRPD3, providing some evidence for downstream crosstalk between DR and dRPD3.

Both alleles of *rpd3* affected *4E-BP* transcription, whereas only one allele affected *Tor* mRNA levels. We therefore asked whether longevity modulation by dRPD3 and DR involved crosstalk mediated by 4E-BP. A hypomorphic *4E-BP* allele reduced *4E-BP* transcription to a level roughly similar to the reduction obtained with *rpd3* mutations. This *4E-BP* allele was shown to reduce longevity, making it likely that we could detect the potential crosstalk. Since the *rpd3* alleles increased longevity but maintained DR-mediated extension, and since the *4E-BP* allele decreased longevity but maintained DR-mediated extension, we also asked whether double mutants would show maintenance of DR-mediated extension. dRPD3-mediated longevity extension was decreased by the presence of a *4E-BP* mutation at all nutrient levels, suggesting distinct effects upon longevity. However, a DR effect was absent in males that are double mutants and was moderately decreased in females that are double mutants. This suggests either that 4E-BP helps mediate the DR effect, or more likely, a protein whose level is regulated by 4E-BP helps mediate the interaction between DR and dRPD3. A recent study examined whether *Drosophila* longevity extension mediated by *chico* mutations was dependent upon 4E-BP [42]; the *chico* gene product is part of the insulin signaling pathway. The longevity phenotype of *chico* heterozygotes was found to be partially dependent upon 4E-BP, but not the longevity phenotype of *chico* homozygotes. They found that 4E-BP-null flies have no altered longevity, but flies that were both 4E-BP null and *chico* heterozygotes had mean life spans intermediate between controls and *chico* heterozygotes (wild type for 4E-BP). Our study and the *chico* study illustrate that longevity regulation is complex, with multiple pathways affecting longevity and crosstalk between the pathways. In addition, longevity regulation seems to be highly sensitive to the dosage of proteins linked to this regulation. In summary, our results show that DR and dRPD3 can have discrete and therefore additive effects upon longevity. However, DR and dRPD3 show some interaction. Additional evidence is presented that implicates 4E-BP in the crosstalk between longevity regulation mediated by nutrients and dRPD3.

## MATERIAL AND METHODS

### Fly strains and maintenance

*rpd3-deficient (rpd3^def24^/TM6,Sb)*, *rpd3*–hypomorphic (*rpd3^P-UTR^/TM3,Sb,Ser*), and control for *rpd3*–hypomorphic (*rpd3^P-1.8^/TM6,Tb*) strains were used in our experiments. The hypomorphic *rpd3^P-UTR^* allele has a P-element inserted in the 5′UTR region of the *rpd3* gene, close to the transcription start site, which affects expression throughout the fly's body [10]. The control *rpd3^P-1.8^* allele has a P-element inserted 1.8 kb upstream from the transcriptional start site, which only decreases expression in the eye [10]. The *rpd3^P-UTR^* and *rpd3^P-1.8^* alleles are derived from the same mutagenesis. The *rpd3^def^* allele was derived by excision of the P-element from *rpd3^P-UTR^* [10]. *Canton S* (*CS*), *yw* and *Thor^k07736^* flies were kindly provided by the Bloomington Stock center. *Thor^k07736^* flies were backcrossed to the *yw* strain for 10 generations to minimize the effects of genetic background.

### Maintenance of aging flies

Flies were collected within 24 hours of eclosion and maintained using standard culture media in plastic vials. They were kept at 25°C in a humidified incubator on a 12 hour light: dark cycle. About 25 males and 25 females are kept together in each vial. For life span studies flies were passed every 2 days up to age 30 days and every day after that and the number of dead flies were counted. The number of flies in each life span study is listed in Tables 1 – 8. Standard corn food was prepared as previously described [43, 44]. The nutrient media used are dilutions (0.1N, 0.3N, 0.5N, 0.7N) or concentrations (1.5N) of an arbitrary normal condition, 1.0N, containing 100 g/L brewer's yeast (MP Biomedicals, Inc.), 100 g/L sucrose (MP Biomedicals, Inc.), 20 g/L agar, and 10 mg/L tegosept. Our nutrient protocol coordinately changes both sugar and protein by varying sucrose and yeast [26, 29]. The concentrations of agar and tegosept are not varied for the various media.

### Genetic crosses

Male *yw* flies were crossed to female *rpd3^def24^/TM6,Sb*. F1 progeny not carrying the *TM6* balancer were used for life span measurements (flies carrying the mutation). F1 progeny carrying the balancer were crossed to each other and F2 progeny not carrying the balancer and not *yw* were used as the life span controls. Male flies homozygous for *Thor^k07736^* (backcrossed into a *yw* genetic background) were crossed to female *rpd3^def24^/TM6,Sb* flies. F1 progeny not carrying the balancer were used for life span measurements (doubly mutant flies). Life span measurements with flies only heterozygous for *Thor^k07736^* were performed with progeny from crosses of *Thor^k07736^* homozygotes and *yw*. For studies using the other *rpd3* alleles, *rpd3^P-UTR^*/*TM3,Sb* or *rpd3^P-1.8^*/*TM6,Sb* were crossed to wild type *CS* and F1 progeny not containing the balancer were used for life span measurements.

### Quantitative PCR (Q-PCR)

Flies were separated by sex and frozen at the appropriate age. Thoraces of *rpd3^P-UTR^* /+ and *rpd3^P-1.8^*/+ flies were dissected since the *rpd3^P-1.8^* allele has reduced expression in the eye. Heads and thoraces were dissected from *rpd3^def^*/+ and +/+ flies since the *rpd3* is reduced throughout the body in *rpd3^def^*/+. RNA was isolated by using the standard Chomczynski protocol and Trizol reagent (Gibco BRL) [44]. cDNA was synthesized from RNA. Using TaqMan primers and the Applied Biosystems Thermal Cycler (AB 7500 System), levels of gene expression were determined. Ankyrin was used as an endogenous control [46].

### Statistical analysis

Statistical analysis of Q-PCR results was done using the Student's T-Test from at least three independent experiments and expressed as P values. Error bars represent the standard errors, and P values are specifically indicated in the figures. Life span data were analyzed by Log-Rank tests using the JMP-11 program (SAS). All survivorship data were censored for the first 10 days. Maximum life span was calculated as the mean life span of the longest surviving 10% of the population.

## SUPPLEMENTAL FIGURES


