# Supplementary material for: RPD3 histone deacetylase and nutrition have distinct but interacting effects on Drosophila longevity
Source: Aging (Albany NY). 2015 Dec 8;7(12):1112–28. doi: 10.18632/aging.100856 (PMC4712336; doi:10.18632/aging.100856)
Supplement: Supplementary file 1 [file aging-07-1112-s001.pdf]

SUPPLEMENTAL FIGURES

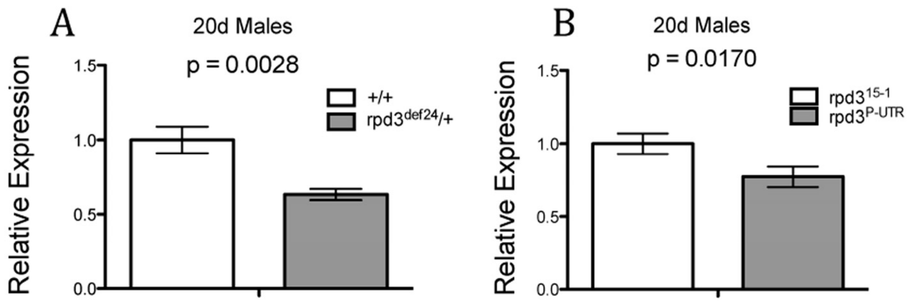

Supplemental Figure 1. The effect of *rpd3* mutations of *rpd3* mRNA levels. (**A**) Q-PCR results showing average *rpd3* mRNA expression in heads and thoraces of 20 day old males, *rpd3*^{def24}*/+* compared to *+/+*. (**B**) Q-PCR results showing average *rpd3* mRNA expression in thoraces of 20 day old males, *rpd3*$^{P\text{-}UTR}$*/+* compared to *rpd3*$^{P\text{-}1.8}$*/+*. 25 heads or thoraces per replicate.

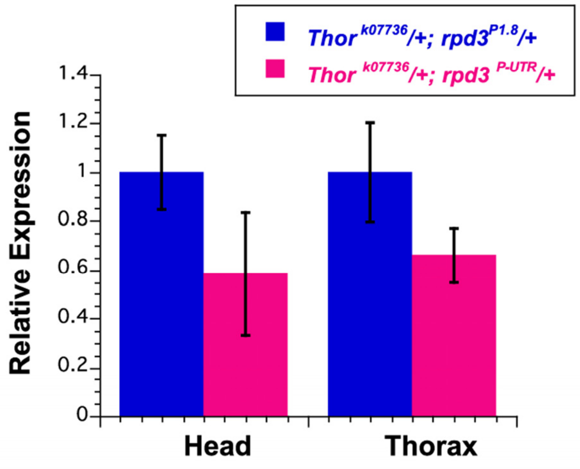

Supplemental Figure 2. Average expression of mRNA for the *4E-BP* gene in three biological replicates isolated from the heads and thoraces of female flies at 10 days of age by Q-PCR. Blue, *Thor*^{k07736}*/+;rpd3*$^{P\text{-}1.8}$*/+* controls, magenta, *Thor*^{k07736}*/+;rpd3*$^{P\text{-}UT}$*R/+*. Graphs are plotted as the means +/- standard errors.
